# Spectrophotometric determination of favipiravir in presence of its acid hydrolysis product

**DOI:** 10.1186/s13065-023-01046-6

**Published:** 2023-09-30

**Authors:** Yasmine Ahmed Sharaf, Mai H. Abd El-Fattah, Heba M. El-Sayed, Maha A. Hegazy

**Affiliations:** 1 Department of Analytical Chemistry, Faculty of Pharmacy, Zagazig University, Zagazig, 44519 Egypt; 2 Pharmaceutical Analytical Chemistry Department, College of Pharmaceutical Sciences and Drug Manufacturing, Misr University for Science & Technology, Giza, 12566 Egypt; 3 Department of Analytical Chemistry, Faculty of Pharmacy, Cairo University, Cairo, 11562 Egypt

**Keywords:** Favipiravir, Acid-induced degradation, Spectrophotometry, Pharmaceutical formulation

## Abstract

Favipiravir (FAV) has been approved as an antiviral drug used in pandemic corona virus to treat covid-19. It has an amide moiety susceptible to hydrolysis and degradation in acid medium. Therefore, four simple, sensitive, and accurate stability indicating spectrophotometric methods have been developed for the determination of FAV in presence of its acid induced degradation product. The first method describes direct determination of FAV at 323 nm. Dual wavelength method was the second developed one for FAV quantitation by recording the absorbance difference at 322.7 and 270 nm. The third method involves using first derivative peak to peak amplitude at 338.0 and 308.0 nm, while difference spectrophotometry was the fourth suggested method, and it was based on recording the spectral changes at 361.3 nm as pH changes. The obtained calibration curves were linear over 4.0–22.0 µg/mL. Accuracy of the suggested procedures ranged from 99.11 to100.06, while precision results were from 0.80 to1.68. The developed methods were used to determine FAV in pure powdered form, laboratory-prepared mixtures with their degradation product, and pharmaceutical formulation without interference from its acidic degradation product.The greenness was assessed based on GAPI and ACREE metric and was found to be compatible and in reconciliation with green analytical chemistry concepts.

**Graphical Abstract:**

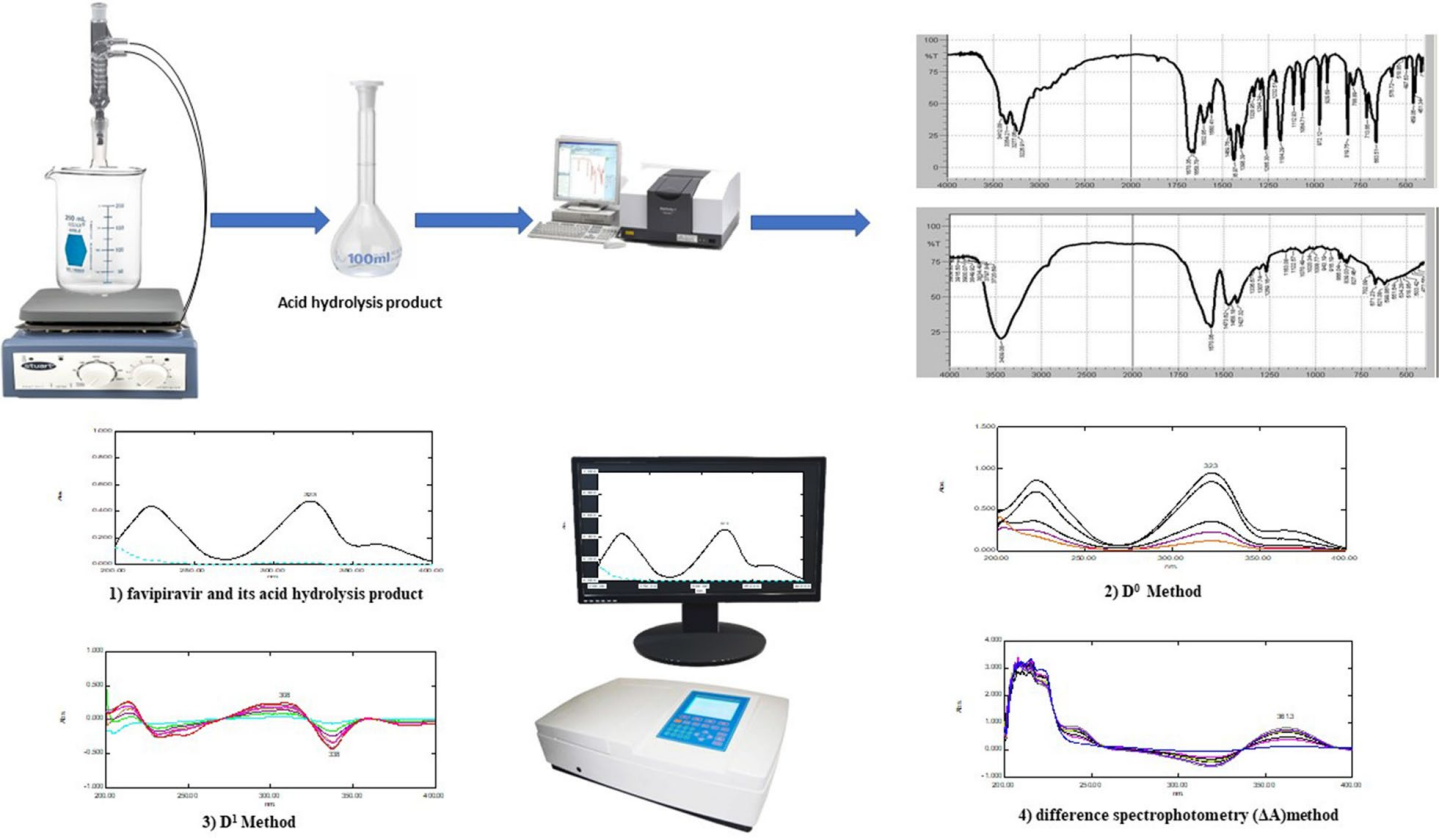

**Supplementary Information:**

The online version contains supplementary material available at 10.1186/s13065-023-01046-6.

## Introduction

**Fig. 1:**
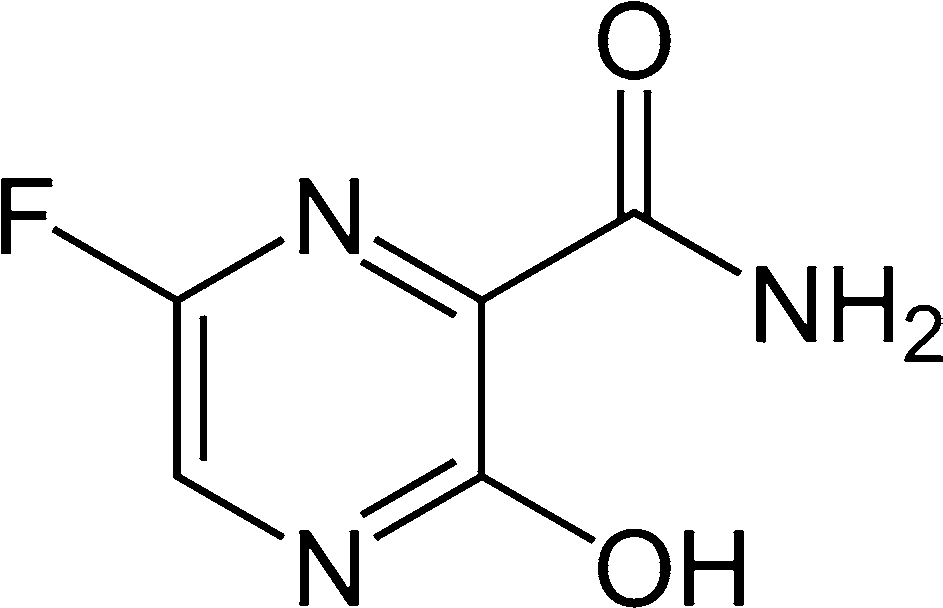
Chemical structure of favipiravir

Globally, there have been more than four hundred million confirmed cases of COVID-19, including about 6 million deaths, reported to the World Head Organization (WHO). COVID-19 (coronavirus disease 2019) is a disease caused by a virus known as SARS-CoV-2. It spreads from infected person to another through breathing out droplets and small particles which contain the virus. Many trials have been done to solve this problem and treat patients. One of the most important and effective drugs that have been discovered and used to treat COVID-19 is Favipiravir (FAV). It was approved to treat novel viruses including Ebola and most recently, COVID-19. FAV is (5-fluoro-2-oxo-1*H*-pyrazine-3-carboxamide), Fig. 1. It is a synthetic purine base analog prodrug that is converted to active favipiravir ribofuranosyl-5B-triphosphate (FAV-RTP) by intracellular phosphoribosylation [1], This active form binds to and hinders RNA dependent RNA polymerase (RdRp), which finally blocks viral transcription and replication.

Stability study of drugs is considered one of the most important ways to confirm drug stability and is a regulatory requirement as well. Forced degradation must be the first step in method development. Hydrolysis is one of the major mechanisms for degradation of biodegradable materials [2]. Identification of primary degradation products as well as unknown impurities is then performed.

The literatures showed several analytical methods for the quantitative determination of FAV including spectrophotometry [3], spectrofluorimetry [4–6], HPLC and LC-UV [7–20], HPTLC [21, 22] and electrochemical methods [23–27] in pharmaceutical formulations or plasma.

In this paper, four spectrophotometric methods were applied for determination of FAV in the presence of its acid-induced degradation product (FAV deg.) in pure form and in pharmaceutical dosage form. The applied methods are Direct, Dual wavelength (DW), difference spectrophotometry (ΔA) and first derivative peak to peak (D^1^). The developed methods are simple, accurate, validated, cost-effective, and can be used for the determination of FAV in the presence of (FAV deg.).

## Experimental

### Instrumentation

Spectrophotometric measurements were performed using Shimadzu 1601 spectrophotometer (Tokyo, Japan), a double beam UV–Vis spectrophotometer assisted with UV Probe software (version 2.51). Scanning was done at 200–400 nm range, with 0.1 nm intervals. PH meter (Jenway) was used for pH adjustment. UV-lamp with short wavelength (254 nm; Deuterium, USA). IR Spectrophotometer: Shimadzu 435 (Kyoto, Japan), sampling was undertaken as potassium bromide disks.

### Materials and reagents

All reagents and solvents utilized during the experiment were of analytical grade. Methanol and Hydrochloric acid (Sigma-Aldrich, Germany while Sodium hydroxide was obtained from VWR Chemicals, US. FAV pure drug (100.03 ± 0.61%) was kindly supplied from Eva Pharm Co. (Cairo, Egypt). Piravafi^®^ tablets (batch no.2132642; Marcyrl Pharmaceutical Industries, Egypt) containing 200.0 mg FAV per tablet were purchased from local market.

### Stock standard solution preparation

A stock standard solution of FAV (100.0 µg/mL) was prepared by dissolving 10.0 mg of FAV in 100.0 mL methanol.

### Acid-induced degradation solution preparation

Acidic degradation of FAV was done to study its stability, according to International Council for Harmonisation of Technical Requirements for Pharmaceuticals for Human Use (ICH) guideline [28], 25 mg of FAV was dissolved in 25.0 mL 1.0 N HCl, refluxed for 1 h in water bath at 100 °C then neutralization was done by 2.0 N NaOH, the volume was completed with a 100.0 ml volumetric flask to obtain 250.0 µg/mL.

Complete degradation was confirmed using the TLC developing system composed of ethyl acetate–methanol-ammonia (2:4:0.1) by volume, Additional file 1: Fig. S1.

### IR sample preparation

For Favipiravir, about 150 mg of the KBR salt was weighted, poured into the mortar then Favipiravir was added, just a small amount on the tip of a spatula. Mixing Favipiravir with KBR salt in a good manner using pestle to ensure obtaining homogenous powder and to avoid obtaining a pellet too sticky which may cause a difficulty in getting it out of that pellet press. Next, add a small amount of homogenous powder into pellet press which consist of three pieces” the short bolt, the large bolt and then the doughnut.” Put the solid sample to be pressed into the pellet to give a thin layer covering the surface of the metal; not little or large quantity to obtain desired pellet not thick or fine which may make a pellet not hold up when pressed it. Support the bottom of the die set into the pellet press. Pressing the solid sample done between the two bolts about five to ten seconds to confirm completely pressing. Opening pellet up, taking the middle part with transparent layer of powder formed after pressing. That transparent nature was important because IR is going to have to pass through that pellet, to get some kind of absorbency inside the machine. That’s explain why it must not be too thick Additional file 2: Fig. S2.

For FAV degradation, after acidic degradation of Favipiravir and neutralization with NaOH, salt and water were formed. Evaporation was done using rotavap after that residue was washed with methanol and evaporated twice to obtain finally the degradation product in a pure solid form, applying on it the same previous procedure as Favipiravir.

## Procedure

### Construction of the calibration curve.

#### Direct spectrophotometric method

FAV working solutions (4.00–22.00 µg/mL) were prepared by accurately transferring aliquot from the stock standard solution using methanol as a diluent. Scanning the prepared solutions was done from 200.0 to 400.0 nm. The quantitative determination of FAV was done at 323.0 nm. The calibration curve was constructed by plotting the absorbance versus the corresponding concentration.

#### Dual wavelength spectrophotometric method (DW)

Using the previously scanned spectra, a calibration curve was constructed by correlating the absorbance difference between 322.7 and 270.0 nm to the corresponding FAV concentrations. At the selected wavelengths FAV deg. has zero difference in absorbance.

#### *First derivative (D*^*1*^*) peak to peak spectrophotometric method*

Using zero order spectra, a manipulation operation was done to obtain their D^1^ derivative spectra with 10 scaling factor and (Δλ = 4). A calibration curve was constructed from D^1^ spectra by plotting sum of FAV peak amplitudes at 338.0 and 308.0 nm versus its corresponding concentrations over a range of 4.0–22.0 µg/mL.

#### Difference spectrophotometric method (ΔA)

In this method, serial dilutions with 1.0 N NaOH were measured against the same drug concentrations in 1.0 N HCL as a blank, the calibration curve was constructed by recording the absorbance of FAV working solutions diluted with 1.0 N NaOH against the same concentrations of FAV diluted with 1.0 N HCl at 361.3 nm in a range of 4.0–22.0 µg/mL.

### Analysis of laboratory prepared mixtures

Mixtures containing different ratios of FAV and FAV Deg. were prepared, then the absorption spectra of these mixtures were recorded. Then the procedures were completed as previously discussed for Direct, DW, D^1^ and ΔA. The concentration of FAV was calculated using the corresponding regression equation for each method.

### Application to a pharmaceutical dosage form

Ten tablets of Piravafi ^®^ were accurately weighed and grinded into fine powder after removing their coats. A quantity of powdered tablets equivalent to 10.0 mg of FAV was accurately weighed and transferred to a 100 mL volumetric flask, 30 mL of methanol were added, sonicated for 15 min, completed to the specified volume with methanol to obtain the final concentration 100 µg/ml then, filtered through a 0.45 um membrane filter. Then the procedures were completed as explained under Construction of the calibration curves.

## Results and discussion

### Acid hydrolysis of FAV

**Scheme1:**
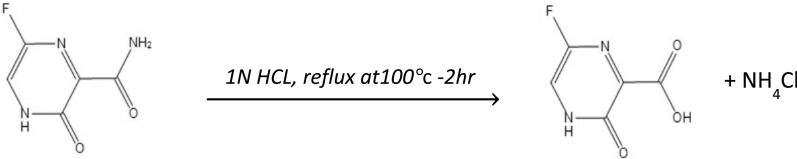
Suggested acidic degradation pathway of Favipiravir

Reviewing the literature, some stability studies described the acidic, alkaline, oxidative, thermal, and photolytic degradation of FAV [8, 17–19]. These studies confirmed that the drug is more liable to acidic degradation. The presence of an amide moiety could be the reason for FAV degradation in acid medium. A reported mechanism suggested the formation of carboxylic acid and ammonium salt of amides on degradation by acid hydrolysis [29] as shown in scheme 1.

IR spectroscopy was utilized to confirm the suggested mechanism. Unsubstituted amides, -CO-NH_2_, were reported to have two NH_2_ stretch bands near 3350 cm^−1^ and 3180 cm^−1^. In addition, the C=O of an amide has stretch band at about 1680–1640 cm^−1^ as well as an NH_2_ deformation band at about 1640–1620 cm^−1^. Identification of carboxylic acid salts can be done by the presence of two nearly equivalent bonds in -CO_2_^−^ group which are intermediate between C=O and C-O. These bonds have a characteristic stretch bands at 1650–1540 and 1450–1360 cm^−1^ and these values vary for different positive counterion [30].

**Fig. 2:**
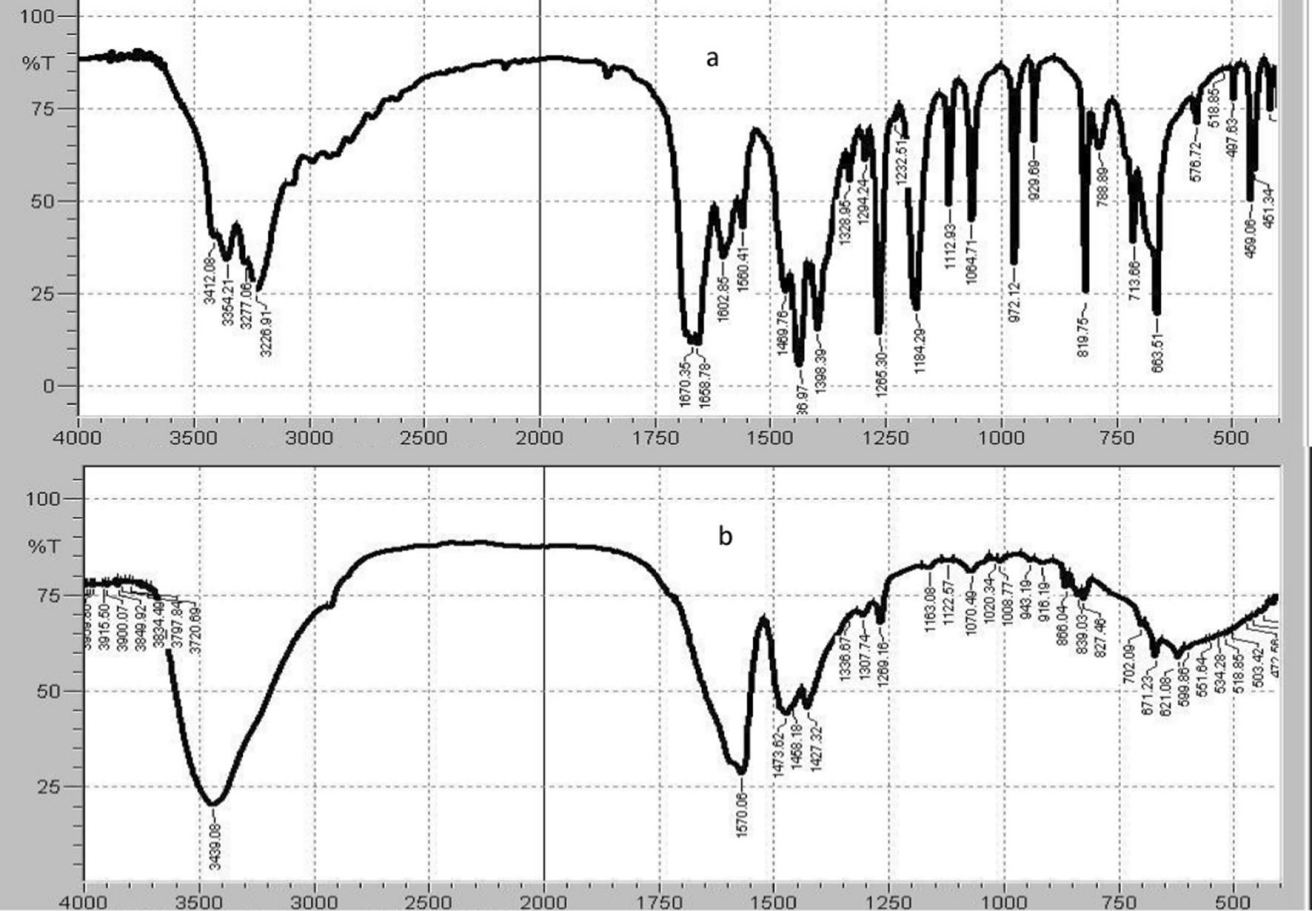
IR spectrum of (**a**) Favipiravir, (**b**) acid-induced degradation product

The IR chart of intact FAV shows two NH_2_ stretch bands at 3354 and 3226 cm^–1^. Meanwhile the stretch band of C=O at 1670 cm^−1^, and the NH_2_ deformation band at 1658 cm^−1^ are clearly noticed (Fig. 2a). On the other hand, Fig. 2b represents the IR chart of FAV deg. in which NH_2_ stretch bands are not found, thus verifying the cleavage of NH_2_ group. Moreover, two bands specific for carboxylate group at 1570 and 1473 cm^−1^ can be noticed. Therefore, the suggested acid hydrolysis mechanism was confirmed.

### Spectrophotometric methods

**Fig. 3:**
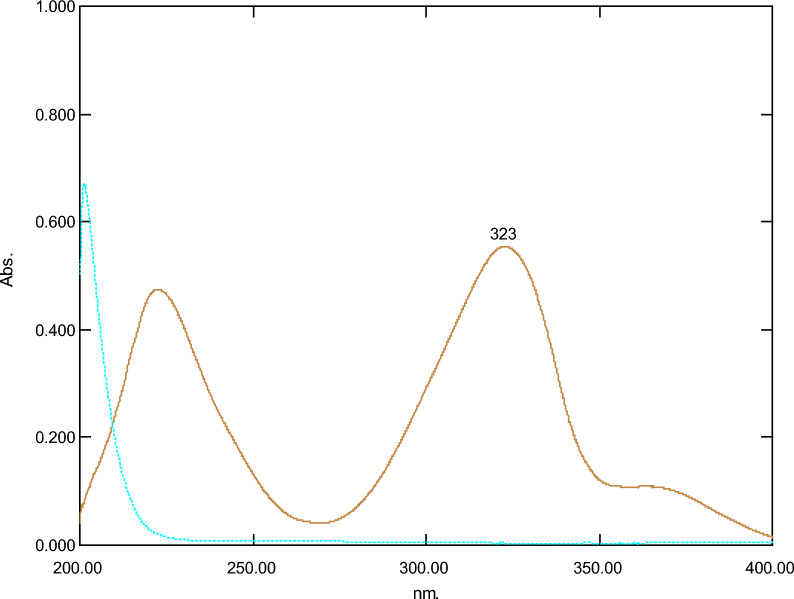
Absorption spectra of 10 µg of favipiravir (—) and 10 µg of its acid-induced degradation product (- - -)

Absorption spectra of FAV and FAV deg. (Figure. 3) showed a partial interference from FAV deg. Therefore, the main goal of this research is to develop simple, selective, cost-effective, and eco-friendly UV-spectrophotometric methods for assessment of FAV in presence of FAV deg. The developed methods include *D*^*0*^, DW, D^1^ and ΔA.

#### *Direct spectrophotometric method (D*^*0*^*)*

**Fig. 4:**
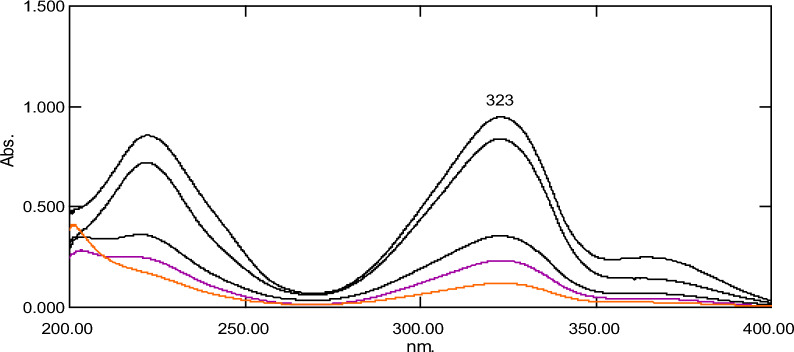
Absorption spectra of laboratory prepared mixtures of favipiravir (2–18 µg/mL) and its acidic degradation product (2–18 µg/mL) in methanol at 323 nm

It is an important, smart, and easy method for determination of the concentration of FAV in presence of FAV deg. Figure 4 presents D^0^ spectra of FAV over the range of 4.0–22.0 µg/mL.

#### Dual wavelength spectrophotometric method (DW)

DW is an accurate, easy method, with no need for software programs which make it simple to be applied. For the elimination of this overlapping, two wavelengths in the mixture spectra were chosen in a way that there is an absorbance difference between the chosen wavelengths directly proportional to FAV concentration while for FAV degradation the difference equals to zero. FAV concentration was calculated using the corresponding regression equation, Table 1.

**Table 1:** Regression method validation parameters data of the proposed spectrophotometric methods for favipiravir determination

	Methods			
Parameter	Zero order	DW	D^1^	ΔA
Linearity range (µg/mL)	4.00–22.00	4.00–22.00	4.00–22.00	4.00–22.00
Slope	0.0567	0.0526	0.0443	0.0471
Intercept	0.0756	0.0696	0.0876	0.0159
Determination coefficient (r^2^)	0.9995	0.9998	0.9999	0.9997
Accuracy (mean ± SD)	100.06 ± 1.52	99.47 ± 0.77	100.01 ± 1.11	99.11 ± 1.17
LOD (µg/mL)	0.578	0.347	0.301	0.483
LOQ (µg/mL)	1.929	1.158	1.003	1.612
Intra-day precision RSD%^a^	0.802	1.051	0.811	0.949
Inter-day precision RSD%^a^	1.131	1.144	1.027	1.053

^a^Average of RSDs of three concentrations in triplicate analysis, the concentrations were as follows (10.0, 14.0, 18.0 μg/mL)

#### *First derivative (D*^*1*^*) peak to peak spectrophotometric method*

**Fig. 5:**
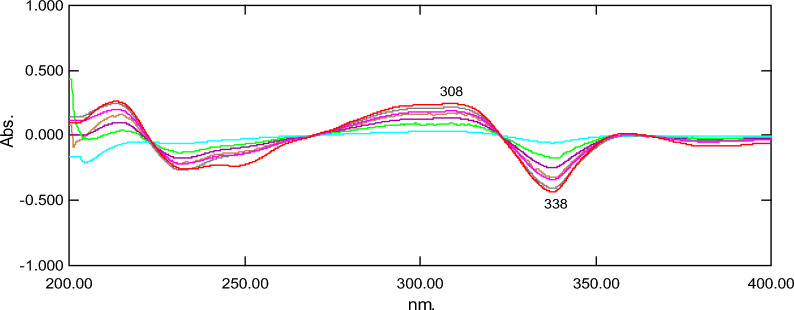
First derivative spectra for mixtures of favipiravir (2–18 µg/mL) and its acidic degradation product (2–18 µg/mL)

This method is simple, easy to apply for resolving FAV and FAV deg. This method basically depends on the derivatization of the stored spectra of FAV&FAV deg. D^1^ spectra of FAV and FAV deg. is presented in Fig. 5.

#### Difference spectrophotometric method (ΔA)

**Fig. 6:**
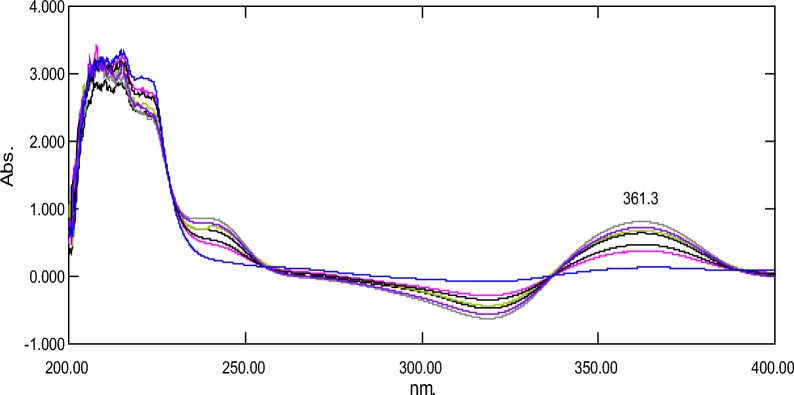
Laboratory prepared mixtures of favipiravir (2–18 µg/mL) and its acidic degradation product (2–18 µg/mL) in sodium hydroxide against hydrochloric acid

Another simple, easy, and accurate method was applied for determination of concentration of FAV in presence of FAV deg. This method was based on the recording induced effect of pH on absorption spectra of FAV at 361.3 nm where FAV has higher absorbance in NaOH than HCl (Fig. 6). Table 1 shows the linear regression equation of the suggested method.

Favipiravir contains a phenolic group which is considered as weak acid so can react with a base as NaOH which is strong base, forming sodium phenoxide and water. Sodium phenoxide is a moderately strong base increase pH leading to bathochromic shift [31, 32]. Sodium phenoxide contains negative charge on oxygen atom. The delocalization of negative charge on the oxygen atom over the aromatic ring (conjugation) as compared to the lone pairs which results in a lower energy gap between HOMO and LUMO which mean more stabilization leading to higher wavelength (bathochromic shift) as they are inversely proportional to each other that’s explain and summarize why change in wavelength maxima to 361.3 nm.

### Methods validation

According to ICH guidelines [33], the proposed UV methods were suggested to confirm methods validation.

### Linearity and range

The linearity of the suggested methods was assessed by analyzing five concentrations of FAV over a range of 4.0–22.0 µg/mL. Absorbance was recorded by scanning three replicates of each concentration at 200–400 nm using methanol as a blank in the first three methods, while the last method using drug in1.0N HCL as blank. The results showed good linear relationships according to the computed correlation coefficient values listed in Table 1.

### Limit of detection [LOD] and limit of quantification [LOQ]

Values of LOD and LOQ were computed to confirm and evaluate method sensitivity (Table 1). LOD and LOQ were calculated using following equations:

$${\text{LOD }} = \, \left( {{3}.{3 }*{\text{SD}}} \right)/{\text{S}}$$

$${\text{LOQ }} = \, \left( {{1}0 \, *{\text{SD}}} \right)/{\text{S}}$$

where SD is the standard deviation of y-intercept and S is the slope of the calibration graph.

### Accuracy and precision

The accuracy of our methods was evaluated by applying these methods for determination of five different concentrations of FAV in triplicate. FAV concentrations were calculated from the corresponding regression equations of the developed methods after that percentage recoveries determination. Results obtained were within the acceptable limits.

Intra-day and inter-day precision were studied by analysis of three different concentrations of FAV 3 times on the same day and on three consecutive days, respectively. The percentage relative standard deviation (RSD%) values were calculated, and satisfactory results were obtained (Table 1).

### Specificity

Different ratios of laboratory mixtures of FAV and FAV deg. were prepared within their linearity range and analyzed to evaluate method specificity. Results confirmed that FAV can be quantified in presence of FAV deg. without any interference from degradation product up to 60% degradation product in laboratory mixture (Table 2).

**Table 2:** Determination of favipiravir in presence of its acid-induced degradation product in laboratory prepared mixtures with proposed spectrophotometric methods

Conc (µg/mL)		Direct	DW	D^1^	ΔA
FAV	Deg	Recovery%^a^			
18.00	2.00	99.90	100.40	99.27	100.25
16.00	4.00	100.70	100.71	99.97	99.47
14.00	6.00	99.47	100.16	98.94	98.58
10.00	10.00	99.75	99.92	100.81	99.77
8.00	16.00	100.88	99.48	99.77	105.07
6.00	14.00	116.87	123.76	130.02	120.97
2.00	18.00	172.48	166.92	194.80	152.76

^a^Average of three determinations

### Application of these methods to pharmaceutical dosage forms

The developed methods were valid for the quantitative determination of FAV in pharmaceutical formulations. Results of the suggested procedures showed high recoveries % as summarized in Table 3.

**Table 3:** Statistical analysis of proposed and reported methods for the analysis of Pirafavi^®^ tablets

Parameters	Reported method	Zero order	DW	D^1^	ΔA
Mean recovery %	102.35	102.03	100.01	99.41	102.42
Variance	17.88	13.67	9.33	11.37	51.99
n	5	7	6	6	7
Students t-test	–	0.135 (1.895)^a^	1.033 (2.262)^a^	1.160 (2.262)^a^	0.023 (2.228)^a^
F-test	–	1.31 (5.19)^a^	1.91 (6.16)^a^	1.57 (6.16)^a^	2.91 (5.19)^a^

^a^Tabulated t- and F- values

### Statistical analysis

Statistical comparison of the results obtained from the developed and reported method [8] was performed, where Student’s t-test and variance ratio F-test showed no significant difference between the two methods as shown in (Table 3) v. In addition, the proposed methods were compared using a one-way ANOVA test. Results showed no significant variations between the methods as the calculated F-values did not exceed the critical one (Table 4).

**Table 4:** One-way ANOVA results for determination of favipiravir using the proposed spectrophotometric methods

Source of variation	Sum of squares	Degree of freedom	Mean of squares	*F*-value	*P*-value	Critical F
Between groups	42.11713	4	10.52928	0.462519	0.762489	2.776289
Within groups	546.362	24	22.76508			
Total	588.4791	28				

### Greenness assessment

The greenness of the four developed spectrophotometric methods were assessed using the Green Analytical Procedure Index (GAPI) and AGREE metric. GAPI is represented with three colors: green, yellow and red. It deals with and takes into consideration all parts of the process from sample collection to final products and wastes. It indicates hazards and safety of reagent and solvents used [34]. AGREE is another method used to green assessment.it is a clock shaped which divided into 12 sections each one represents one factor of assessment. It also contains three colors green, yellow and red. Score range from 0 to 1 [35].

The developed methods were assessed using both the GAPI and AGREE tools (Table 5).

**Table 5:** Green assessments of the developed spectrophotometric methods

	Zero order	DW	D^1^	ΔA
AGREE				
GAPI				

Comparing the results shown in Tables 1 and 5, the first three methods were greener, but the first method (zero-order) was the most accurate and simplest one.

## Conclusion

The developed methods were simple, rapid, precise, accurate, and easy to apply for routine analysis in the laboratory. They were successfully used to determine FAV in presence of up to 60% FAV deg. The developed procedures were in good agreement with each other and with the reported method. The greenness of validated methodologies was assessed and compared with each other showed that zero-order was the most simple, accurate and green method.

### Supplementary Information

**Additional file 1: S1.** Separation of A) FAV and B) acid-induced degradation product using mobile phase ethyl acetate–methanol-ammonia (2:4:0.1, v/v).

**Additional file 2: S2.** Calibration curves (a) Zero-order method (b) DW (c) D1 (d) ΔA Linearity range (4-22 µg/ml).

## Data Availability

Data will be available on request.

## References

[1] Furuta Y, Komeno T, Nakamura T (2017). Favipiravir (T-705), a broad spectrum inhibitor of viral RNA polymerase. Proc Jpn Acad Ser B Phys Biol Sci.

[2] Lyu S, Untereker D (2009). Degradability of polymers for implantable biomedical devices. Int J Mol Sci.

[3] Jyothi BJ, Kavya RV (2021). Ultraviolet spectrophotometric method development for estimation of new antiviral repurposing drug favipiravir. Asian J Pharm Clin Res.

[4] El Sharkasy ME, Tolba MM, Belal F, Walash M, Aboshabana R (2022). Quantitative analysis of favipiravir and hydroxychloroquine as FDA-approved drugs for treatment of COVID-19 using synchronous spectrofluorimetry: application to pharmaceutical formulations and biological fluids. Luminescence.

[5] El-Awady M, Elmansi H, Belal F (2022). Insights on the quantitative concurrent fluorescence-based analysis of anti-COVID-19 drugs remdesivir and favipiravir. J Fluoresc.

[6] Megahed SM, Habib AA, Hammad SF, Kamal AH (2021). Experimental design approach for development of spectrofluorimetric method for determination of favipiravir; a potential therapeutic agent against COVID-19 virus: application to spiked human plasma. Spectrochim Acta Part A: Mol Biomol Spectrosc.

[7] Bulduk I (2021). HPLC-UV method for quantification of favipiravir in pharmaceutical formulations. Acta Chromatogr.

[8] Marzouk HM, Rezk MR, Gouda AS, Abdel-Megied AM (2022). A novel stability-indicating HPLC-DAD method for determination of favipiravir, a potential antiviral drug for COVID-19 treatment; application to degradation kinetic studies and in-vitro dissolution profiling. Microchem J.

[9] Mikhail IE, Elmansi H, Belal F, Ehab Ibrahim A (2021). Green micellar solvent-free HPLC and spectrofluorimetric determination of favipiravir as one of COVID-19 antiviral regimens. Microchem J.

[10] Duse PV, Baheti KG (2021). Bioanalytical method development and validation for the determination of favipiravir in spiked human plasma by using RP-HPLC. J Pharm Res Int.

[11] Abdallah IA, El-Behairy MF, Ahmed RM, Fayed MA (2022). The anti-COVID-19 drug favipiravir: degradation, method development, validation, NMR/LC–MS characterization, and in-vitro safety evaluation. Chem Pap.

[12] Aydinoglu S, Bozyel M (2022). Favipiravir determination in pharmaceutical formulation via HPLC chromatographic approach. Iran J Chem Chem Eng.

[13] Balu PA, Paresh MS (2021). Stability-indicating RP HPLC method development for estimation of favipiravir in bulk and pharmaceutical dosage form. World J Pharm Res.

[14] Emam AA, Abdelaleem EA, Abdelmomen EH, Abdelmoety RH, Abdelfatah RM (2022). Rapid and ecofriendly UPLC quantification of remdesivir, favipiravir and dexamethasone for accurate therapeutic drug monitoring in Covid-19 patient’s plasma. Microchem J.

[15] Kalshetti M, Adlinge SG (2022). Development and validation of HPLC method for quantification of favipiravir in tablet. Res J Pharm Technol.

[16] Onmaz DE, Abusoglu S, Onmaz M, Yerlikaya FH, Unlu A (2021). Development and validation of a sensitive, fast and simple LC-MS/MS method for the quantitation of favipiravir in human serum. J Chromatogr B.

[17] Vemuri DK, Gundla R, Konduru N, Mallavarapu R, Katari NK (2022). Favipiravir (SARS-CoV-2) degradation impurities: identification and route of degradation mechanism in the finished solid dosage form using LC/LC–MS method. Biomed Chromatogr.

[18] Gökce S, Höl A, Bulduk I (2021). Development and validation of UPLC-MS/MS method for obtaining favipiravir tablet dosage form and evaluation of its behavior under forced conditions. J Pharm Res Int.

[19] Nazifa Sabir Ali S, Mobina L, Mehfuza M, Seema P, Ahmed A, Khan GJ (2021). Analytical method development and validation and forced degradation stability-indicating studies of favipiravir by RP-HPLC and UV in bulk and pharmaceutical dosage form. J Pharm Res Int.

[20] Rezk MR, Badr KA, AbdelNaby NS, Ayyad MM (2021). A novel, rapid and simple UPLC-MS/MS method for quantification of favipiravir in human plasma: application to a bioequivalence study. Biomed Chromatogr.

[21] Noureldeen DA, Boushra JM, Lashien AS, Hakiem AFA, Attia TZ (2022). Novel environment friendly TLC-densitometric method for the determination of anti-coronavirus drugs “remdesivir and favipiravir”: green assessment with application to pharmaceutical formulations and human plasma. Microchem J.

[22] Saraya RE, Deeb SE, Salman BI, Ibrahim AE (2022). Highly sensitive high-performance thin-layer chromatography method for the simultaneous determination of molnupiravir, favipiravir, and ritonavir in pure forms and pharmaceutical formulations. J Sep Sci.

[23] Allahverdiyeva S, Yunusolu O, Yardm Y, entrk Zh (2021). First electrochemical evaluation of favipiravir used as an antiviral option in the treatment of COVID-19: a study of its enhanced voltammetric determination in cationic surfactant media using a boron-doped diamond electrode. Anal Chim Acta.

[24] Mehmandoust M, Khoshnavaz Y, Tuzen M, Erk N (2021). Voltammetric sensor based on bimetallic nanocomposite for determination of favipiravir as an antiviral drug. Microchim Acta.

[25] Mohamed MA, Eldin GMG, Ismail SM, Zine N, Elaissari A, Jaffrezic-Renault N, Errachid A (2021). Innovative electrochemical sensor for the precise determination of the new antiviral COVID-19 treatment favipiravir in the presence of coadministered drugs. J Electroanal Chem.

[26] Galal A, Ahmed YM, Ahmed MH, Atta NF (2021). Electrochemistry and determination of an antiviral drug at ionic liquids crystals-carbon nanotubes modified glassy carbon electrode. J Electrochem Soc.

[27] Mohamed MA, Eldin GM, Ismail SM, Zine N, Elaissari A, Jaffrezic-Renault N, Errachid AJJoEC (2021). Innovative electrochemical sensor for the precise determination of the new antiviral COVID-19 treatment favipiravir in the presence of coadministered drugs. J Electroanal Chem.

[28] ICH harmonised tripartite guideline, Stability testing of new drug substances and products Q1A (R2). In: International Conference on Harmonisation: 2003.

[29] O'Connor C (1970). Acidic and basic amide hydrolysis. Q Rev Chem Soc.

[30] Lin-Vien D, Colthup NB, Fateley WG, Grasselli JG (1991). The handbook of infrared and Raman characteristic frequencies of organic molecules.

[31] Fountaine J, Joshipura P, Keliher P, Johnson J (1974). New ultraviolet ratio spectrophotometric system for the determination of trace amounts of phenolic compounds. Anal Chem.

[32] Fountaine JE, Joshipura PB, Keliher PN, Johnson JD (1975). Determination of pentachlorophenol by ultraviolet ratio spectrophotometry. Anal Chem.

[33] ICH harmonised tripartite guidelineQ2 (R1) (2005). Validation of analytical procedures: text and methodology.

[34] Płotka-Wasylka J (2018). A new tool for the evaluation of the analytical procedure: green analytical procedure index. Talanta.

[35] Pena-Pereira F, Wojnowski W, Tobiszewski M (2020). AGREE—analytical greenness metric approach and software. Anal Chem.

