# Supplementary material for: Spectrophotometric determination of favipiravir in presence of its acid hydrolysis product
Source: BMC Chem. 2023 Sep 30;17(1):129. doi: 10.1186/s13065-023-01046-6 (PMC10542695; doi:10.1186/s13065-023-01046-6)
Supplement: Supplementary file 1 — Additional file 1: S1. Separation of A) FAV and B) acid-induced degradation product using mobile phase ethyl acetate–methanol-ammonia (2:4:0.1, v/v). [file 13065_2023_1046_MOESM1_ESM.docx]

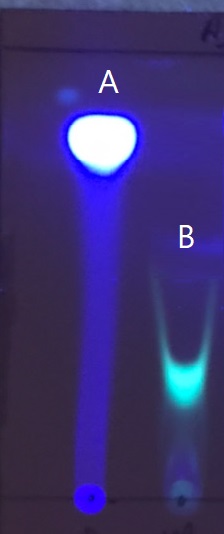


**S1**. Separation of A) FAV and B) acid-induced degradation product using mobile phase ethyl acetate–methanol-ammonia (2:4:0.1, v/v)
