# Supplementary figures and images for: Spectrophotometric determination of favipiravir in presence of its acid hydrolysis product
Source: BMC Chem. 2023 Sep 30;17(1):129. doi: 10.1186/s13065-023-01046-6 (PMC10542695; doi:10.1186/s13065-023-01046-6)

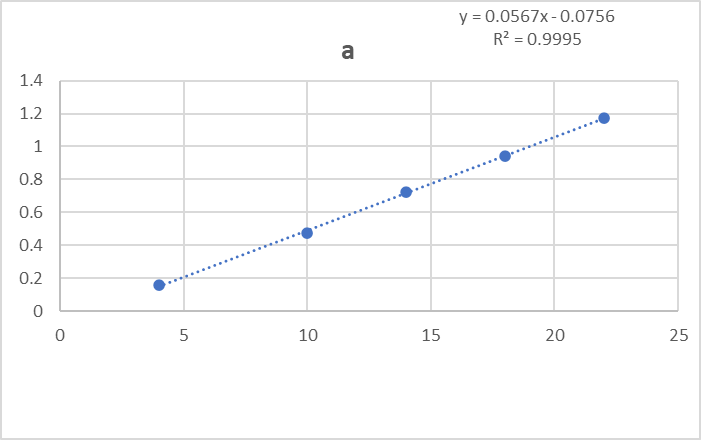


**S2. calibration curves (a) Zero-order method (b) DW (c) D1  (d) ΔA Linearity range (4-22 µg/ml)**


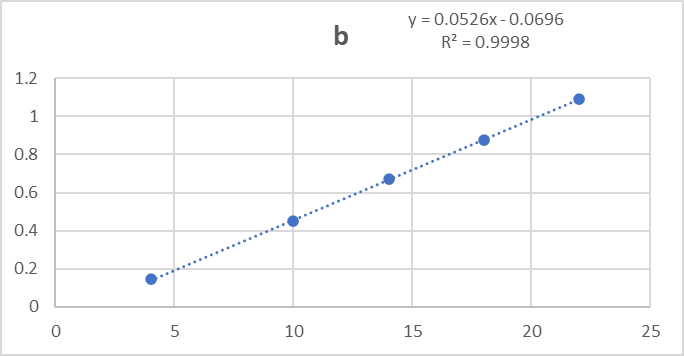

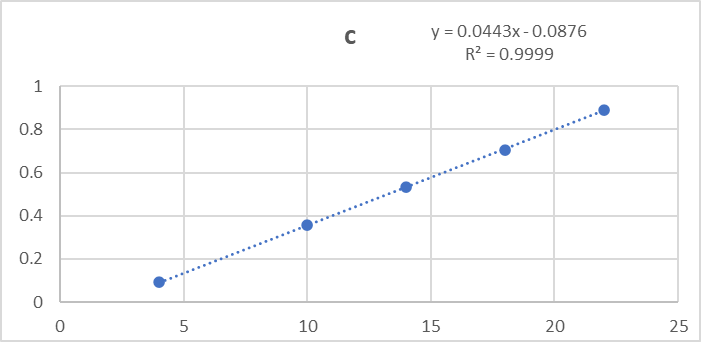

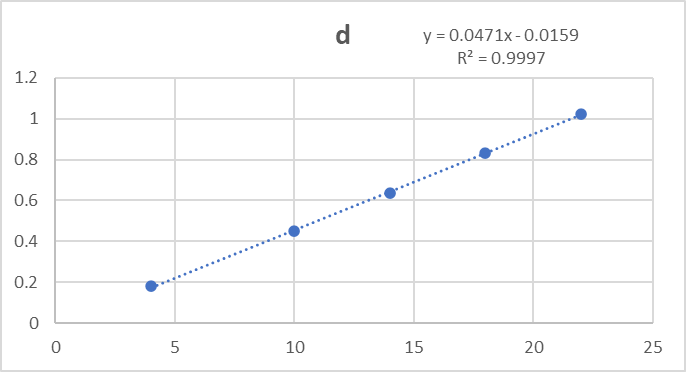

Supplement: Supplementary file 2 — Additional file 2: S2. Calibration curves (a) Zero-order method (b) DW (c) D1 (d) ΔA Linearity range (4-22 µg/ml). [file 13065_2023_1046_MOESM2_ESM.docx]
